# Assessment of late cardiotoxic effects in patients treated for cancer in childhood

**DOI:** 10.1002/cam4.4564

**Published:** 2022-02-15

**Authors:** Vladimír Kincl, Roman Panovský, Tomáš Kepák, Viera Bajčiová, Veronika Bednárová, Lukáš Opatřil, Jan Máchal

**Affiliations:** ^1^ Department of Internal Medicine/Cardiology St. Anne's University Hospital, Faculty of Medicine, Masaryk University Brno Czech Republic; ^2^ International Clinical Research Center St. Anne's University Hospital Brno Czech Republic; ^3^ Department of Children's Oncology University Hospital Brno, Masaryk University Brno Czech Republic; ^4^ Department of Pathological Physiology, Faculty of Medicine Masaryk University Brno Czech Republic

## Abstract

**Background:**

The most frequent chemotherapeutic regimens used in children's cancer contain anthracyclines, which may have potentially cardiotoxic effects even at a later point in time. Echocardiography including tissue Doppler imaging is well‐proved noninvasive method to assess cardiac function.

**Objectives:**

To assess the late cardiotoxic effects in young adults treated for various cancer types in childhood.

**Methods:**

Ninety‐eight young adults who were treated for childhood cancer (CCS), median time 12.1 years after finishing the therapy, were comprised into study. All subjects underwent echocardiographic examination including tissue Doppler imaging. The echocardiographic parameters were compared to 57 healthy age‐matched subjects. The CCS group also underwent 24‐h ECG Holter monitoring to reveal any arrhythmias.

**Results:**

The subjects in CCS group had lower *E*′ velocities from lateral (15.6 vs. 17.6 cm/s) and septal part (12.2 vs. 14.4 cm/s) of mitral annulus (*p* for both <0.001) and also lower *S*′ velocity from the tricuspid annulus (12.4. vs. 13.3 cm/s, *p* = 0.013) and tricuspid annulus peak systolic excursion (TAPSE; 22.2 vs. 23.9 mm, *p* = 0.017). There were no significant differences in left ventricular systolic velocities or ejection fraction. The occurrence of arrhythmias did not differ among subgroups of CCS with or without anthracycline therapy.

**Conclusion:**

Childhood cancer survivors had subclinical cardiac impairment in comparison with healthy controls, particularly in left ventricular diastolic function.

Graphical AbstractThe aim was to assess the late cardiotoxic effects in young adults treated for various cancer types in childhood using echocardiography and 24‐h ECG Holter monitoring.

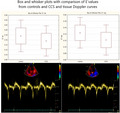

## 1 INTRODUCTION

The therapy of cancer in children has experienced great progress in the last two decades. The development of new drugs and therapeutic procedures has improved the prognosis and quality of life of patients. Nevertheless, cardiotoxic medication, mainly anthracyclines, still play a key role in cancer treatment in children.^1^, ^2^ The cumulative dose of 300 mg/m^2^ has been reported as a risk for heart failure development, but impaired cardiac function can be found in doses less than 300 mg/m^2.^ ^3^, ^4^ Lifelong monitoring of cardiac function is recommended especially in patients treated for acute lymphoblastic leukemia (ALL).^5^ There is no clear evidence of the most appropriate assessment of subclinical cardiac dysfunction in patients treated with lower anthracycline doses. Several studies using echocardiography and spiroergometry did not find significant changes in cardiac function or peak oxygen uptake.^6^, ^7^, ^8^, ^9^, ^10^ On the other hand, Christiansen et al. found lower values of the *E*′ wave in the mitral anulus tissue Doppler imaging and peak oxygen uptake, but both variables only in the anthracycline‐treated subgroup.^11^ We suggest the use of tissue Doppler echocardiography as the first line method to detect subclinical impairment of cardiac function is the best way in patients after potentially cardiotoxic treatment, because it is a well proved, noninvasive, reliable, and simple method to assess changes in global cardiac function. Long‐term occurrence of arrhythmias has not been studied in detail so far, however, Benjanuwattra et al.^12^ published a review analysis of clinical and experimental studies about pro‐arrhythmogenic effect of doxorubicin during the administration period. The purpose of this study is to assess the late cardiotoxic effects including subclinical heart impairment and presence of arrhythmias using tissue Doppler echocardiography and 24‐h ECG Holter monitoring in adults who underwent chemotherapy for cancer in childhood.

## 2 PATIENTS AND METHODS

### 2.1 Study group

One hundred and six adult subjects were initially enrolled into the study. All subjects gave written informed consent before enrollment and the study was approved by the institutional ethic committee (St. Anne's University Hospital, registration number 15V/2018).Subsequently, eight patients were excluded, one for cardiac transplantation before final enrollment and seven for unsatisfactory quality of echocardiographic images. The final number of 98 subjects (68 males)were included in the analysis. All were young adults, mean age 24.2 years, who underwent treatment for various types of cancer in childhood in the Department of Children's Oncology at Brno University Hospital in Brno, Czech Republic between 1978 and 2013.

### 2.2 Echocardiography and Holter ECG

**FIGURE 1:**
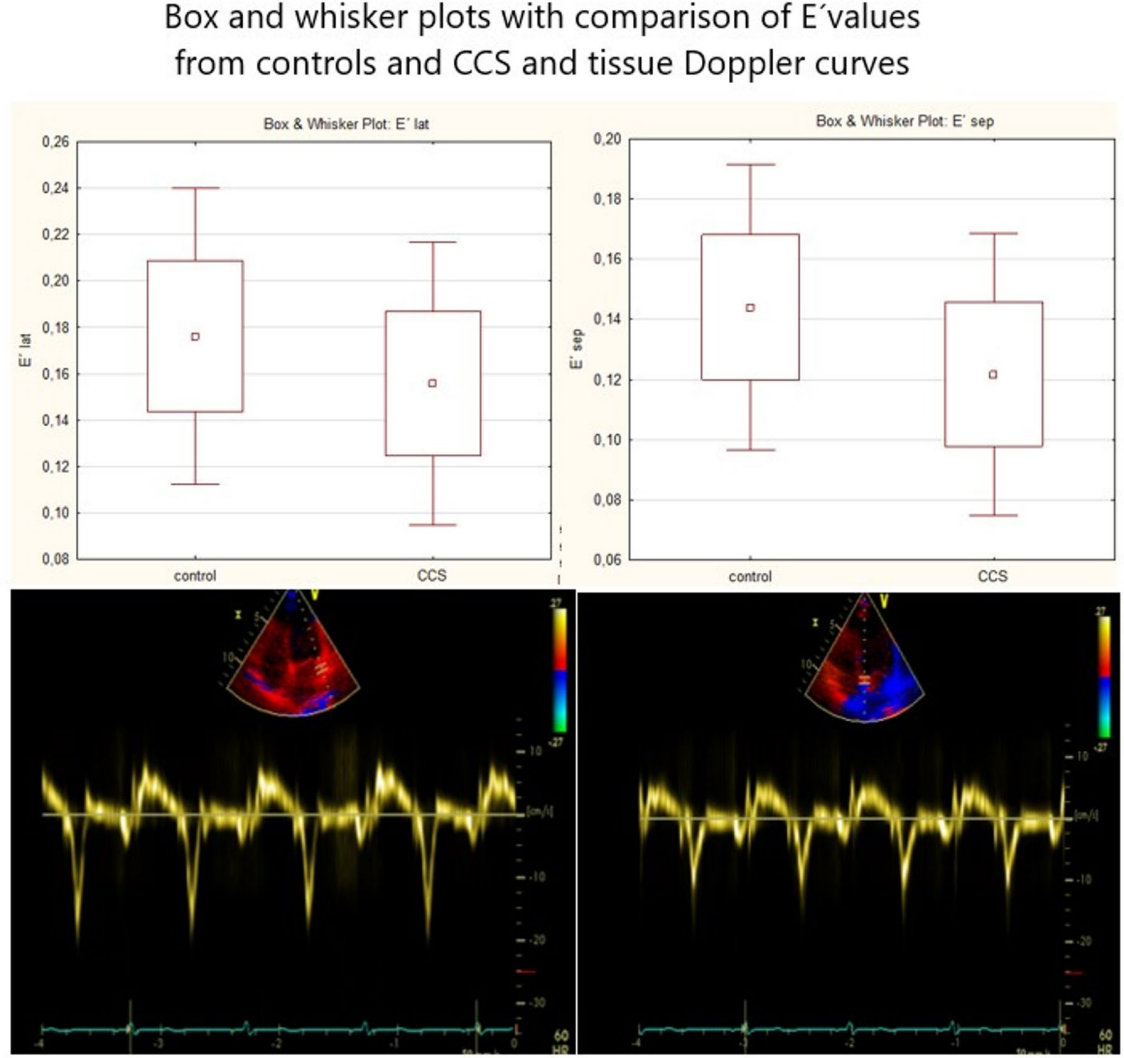
Box and whisker plots with comparison of mean *E*′ values of CCS and controls from lateral (upper left) and septal part of mitral annulus (upper right) and example images of tissue Doppler curves from lateral (lower left) and septal part of mitral annulus (lower right)

All subjects underwent clinical interview and examination focused on potential symptoms of cardiotoxicity such as dyspnea, chest discomfort, ankle swelling, and palpitations. The 24‐h Holter ECG device (MARS, GE Healthcare) was then connected, and patients were asked to have standard daily regimen. The following day the Holter device was disconnected and the record was checked for significant or inappropriate tachy‐ or bradycardia, supraventricular and ventricular arrhythmias, and conduction disturbances. Echocardiography was performed on the Vivid 9 (GE Healthcare), using parasternal and apical views. The heart dimensions were measured and the left ventricular ejection fraction was calculated according to Simpson's rule. Valve morphology and flow characteristics were also assessed for exclusion of significant valve disease. Tissue Doppler parameters were obtained from the apical four‐chamber view, the peak systolic (*S*′), peak early diastolic (*E*′), and late diastolic (*A*′) velocities were measured from the lateral and septal part of the mitral annulus (Figure 1) and lateral part of the tricuspid annulus. Three cardiac cycles were measured and the mean value was used, also the average value from the lateral and septal part of the mitral annulus was calculated.

The control group comprised 57 healthy age‐matched volunteers (mean age 24.3 years, *p* = 0.17, NS vs. study group). All control subjects underwent the same echocardiographic examination as the study patients and values were compared mutually. Patients in the study group were also divided into groups with and without anthracycline administration and according to the indexed anthracycline dose (mg/m^2^) below and above the median (200 mg/m^2^). The echocardiographic parameters and frequency of arrhythmias were assessed among the described groups above.

### 2.3 Statistical analysis

Continuous variables (age, echocardiographic, and tissue Doppler data) generally followed a Gaussian distribution as assessed by the Kolmogorov–Smirnov test and visual inspection of histograms, and were compared by the Student's *t*‐test for unpaired samples between childhood cancer survivors (CCS) and controls. In a subgroup analysis, the CCSs were further divided according to the use of anthracyclines in their medical history into groups with and without anthracycline therapy, and the abovementioned variables were again compared by the Student's *t*‐test; further, the number of supraventricular and ventricular premature beats, as recorded by Holter ECG, was compared using the Mann–Whitney *U* test. The value of *α* = 0.05 was used as the level of statistical significance throughout the analysis.

## 3 RESULTS

The final group consist of 98 patients with complete echocardiographic and ECG Holter data, mean age 24.2 years, with 84 (86%) who were treated by anthracyclines. The mean anthracycline dose was 195 mg/m^2^.Radiotherapy underwent 63 (64%) of the CCS. The most frequent diagnoses were Hodgkin lymphoma (*N* = 30, 29.4%), acute lymphoblastic leukemia (*N* = 16, 15.7%), and the non‐Hodgkin lymphomas (*N* = 14, 13.7%) The mean time since cessation of the therapy was 12.1 years. The complete characteristics of the study group are presented in Table 1, with a summary of diagnoses in Table 2. Regarding echocardiographic parameters, there were no significant differences in left ventricular ejection fraction (LVEF), end‐diastolic and end‐systolic diameters, right ventricular diameter, left ventricular wall thickness, and peak systolic velocities from the lateral and septal part of the mitral annulus, between patients and controls. The CCS had significantly lower peak diastolic velocities from the lateral and septal part of the mitral annulus (15.6 vs. 17.6 cm/s and 12.1 vs. 14.4 cm/s respectively, *p* for both <0.001), tricuspid annulus (13.9 vs. 15.5 cm/s, *p* < 0.001), peak systolic velocity from the tricuspid annulus (12.4 vs. 13.3 cm/s, *p* < 0.013), and TAPSE (22.2 vs. 23.9 mm, *p* < 0.017). Comparison of echocardiographic parameters between CCS and controls are presented in Table 3. The occurrence of arrhythmias was low in the patient group, the mean count of premature supraventricular and ventricular complexes was 17 and 78 per 24 h respectively, no severe arrhythmias such as ventricular tachycardia or atrial fibrillation were recorded. Division of patients into groups of without anthracycline therapy, below median dose (<200 mg/m^2^) and above median dose did not show any significant differences in arrhythmia occurrence or echocardiographic parameters (Table 4).

**TABLE 1:** Basic characteristics of patients’ cohort

Subjects, *N*	98
Males, *N* (%)	65 (66.3%)
Mean age ± SD (years)	24.3 ± 5.4
Body mass index, mean ± SD	22.9 ± 4.1
Mean anthracycline dose ± SD (mg/m^2^)	195.2 ± 121
Time since cessation the cancer therapy, mean ± SD (years)	12.1 ± 5.8
Age at time of diagnosis of cancer, mean (years)	11.8 ± 5.1

Abbreviations: SD, standard deviation.

**TABLE 2:** Types of cancer disorders and their frequency in the study cohort

Type of cancer	*N* (%)
Hodgkin lymphoma	29 (29.6%)
Acute lymphoblastic leukemia	16 (16.3%)
Non‐Hodgkin lymphomas	14 (14.3%)
Ewing sarcoma	7 (7.1%)
Osteosarcoma	6 (6.1%)
Medulloblastoma	5 (5.1%)
Neuroblastoma	5 (5.1%)
Rhabdomyosarcoma	3 (3%)
Glioma	2 (2%)
Burkitt lymphoma	2 (2%)
Pinealoblastoma	1 (1%)
Testis germinal tumor	1 (1%)
Chondrosarcoma	1 (1%)
Acute myeloid leukemia	1 (1%)
Yolk‐sac tumor	1 (1%)
Embryonal carcinoma	1 (1%)
Wilms'tumor	1 (1%)
Synovial sarcoma	1 (1%)
Malignant hepatal carcinoid	1 (1%)

**TABLE 3:** Comparison of echocardiographic parameters of patient and control group

Parameter	Patients CCS	Controls	*p*
EF (%)	62.3 ± 5.5	64.2 ± 5.1	0.62
DD (mm)	43.2 ± 4.6	43.6 ± 4.7	0.62
DS (mm)	28.5 ± 3.8	28.2 ± 4.2	0.63
IVS (mm)	9.1 ± 1.6	9.4 ± 1.5	0.21
PW (mm)	8.6 ± 1.5	8.9 ± 1.1	0.17
*S*′ sept (cm/s)	8.2 ± 1.4	8.5 ± 1.5	0.14
*S*′lat (cm/s)	11.1 ± 2.6	11.1 ± 2.2	0.99
*E*′ sept (cm/s)	12.2 ± 2.4	14.4 ± 2.4	<0.001
*E*′lat (cm/s)	15.6 ± 3.1	17.6 ± 3.3	<0.001
*E*′ avg (cm/s)	13.8 ± 2.7	15.9 ± 2.7	<0.001
*E*′tric (cm/s)	13.9 ± 3.0	15.5 ± 2.3	<0.001
*S*′tric (cm/s)	12.4 ± 2.0	13.3 ± 2.2	<0.013
TAPSE (mm)	22.2 ± 3.8	23.9 ± 4.6	<0.017

*Note*: Data are presented as mean ± standard deviation.

Abbreviations: DD, left ventricular end‐diastolic diameter; DS, left ventricular end‐systolic diameter; *E*′avg, mean value of the peak early diastolic velocity from lateral and septal part of the mitral annulus; EF, left ventricular ejection fraction; *E*′lat, peak early diastolic velocity from septal part of the mitral annulus; *E*′sept, peak early diastolic velocity from septal part of the mitral annulus; *E*′tric, peak early diastolic velocity of the tricuspid annulus; IVS, interventricular septum thickness; PW, posterior wall thickness; *S*'lat, peak systolic velocity from lateral part of the mitral annulus; *S*'sept, peak systolic velocity from septal part of the mitral annulus; *S*'tric, peak systolic velocity of the tricuspid annulus; TAPSE, tricuspid annulus plain systolic excursion.

**TABLE 4:** Comparison of subgroups of childhood cancer survivors, analysis of variance

Parameter	Without anthracycline therapy	Anthracycline dose below median	Anthracycline dose above median	*p*
*S*'sept (cm/s)	8.1 ± 1.4	8.5 ± 1.5	7.9 ± 1.3	0.10
*S*'lat (cm/s)	10.9 ± 3.2	11.3 ± 2.5	10.9 ± 2.7	0.78
*E*'sept (cm/s)	11.9 ± 3.2	12.5 ± 2.5	12.0 ± 2.2	0.68
*E*'lat (cm/s)	15.1 ± 3.6	16.1 ± 3.2	15.8 ± 3.2	0.81
*E*'avg (cm/s)	13.5 ± 3.2	14.1 ± 3.0	13.9 ± 2.4	0.89
*S*'tric (cm/s)	12.1 ± 2.1	13.0 ± 1.9	12.6 ± 1.8	0.64
*E*'tric (cm/s)	12.7 ± 2.9	14.6 ± 3.6	13.7 ± 3.4	0.12
TAPSE (mm)	20.6 ± 3.6	22 ± 3.2	23.1 ± 4.2	0.09
PSVC (N)	9.7 ± 14.8	24.2 ± 80.1	25.1 ± 79	0.44
PVC (N)	339 ± 1290	2.7 ± 6.2	58.5 ± 265	0.10

Abbreviations: *E*'avg, mean value of the peak early diastolic velocity from lateral and septal part of the mitral annulus; *E*'lat, peak early diastolic velocity from septal part of the mitral annulus; *E*'sept, peak early diastolic velocity from septal part of the mitral annulus; *E*'tric, peak early diastolic velocity of the tricuspid annulus; PSVC, premature supraventricular complexes; PVC, premature ventricular complexes; *S*'lat, peak systolic velocity from lateral part of the mitral annulus; *S*'sept, peak systolic velocity from septal part of the mitral annulus; *S*'tric, peak systolic velocity of the tricuspid annulus.

## 4 DISCUSSION

The long‐term side effects including late cardiotoxicity in CCS have been increasingly studied during recent years, reflecting the growing number of adult CCS. The increased cardiovascular morbidity and mortality in patients treated by anthracyclines have also been well described in many studies.^13^, ^14^ Our results, presented above, show that left ventricular systolic function was not impaired in a heterogeneous group of childhood cancer survivors. Only subclinical deterioration of diastolic function of the left ventricle and borderline lower peak systolic velocity of the tricuspid annulus were found, as assessed by tissue Doppler imaging. The impairment of diastolic function is similar as described in the above mentioned study,^11^ but the authors also reported lower systolic parameters in subgroups with lower doses of anthracycline therapy in comparison to anthracycline‐naïve subjects. This was not observed in our study, even though the median anthracycline dose in our cohort was higher (200 vs. 120 mg/m^2^).A recent study by Sofia et al.^15^ compared 20 CCS and 20 controls and described no differences in biplane LVEF calculated by Simpson's rule and global longitudinal strain (GLS), but using RT‐3D echocardiography demonstrated a significant difference in LVEF.However, the mean values of both CCS and controls were in a normal range and no difference between diastolic velocities was found. The patients in this study received a slightly higher dose of anthracyclines, on the other hand the mean follow‐up period was 6.5 ± years, which was a shorter time than in our patient group. Another recently published paper,^16^ described assessment of cardiac impairment in CCS using cardiac magnetic resonance (CMR) for quantification of systolic function and cardiac fibrosis and echocardiography for measurement of diastolic parameters. The cumulative anthracycline dose in CCS was similar to our data (212 mg/m^2^), however the median time since last administration of anthracyclines was also shorter (9.8 vs 12.1 years). The CCS had a significantly lower LVEF and global circumferential strain on CMR and the indexed myocardial mass and mass/volume ratio in the patient group were decreased as well. The LV extracellular volume was also higher in CCS.In contrast, right ventricular EF did not show significant differences against controls. Tissue Doppler echocardiography demonstrated lower peak diastolic velocities from the lateral and septal part of the mitral annulus. The assessment of right ventricular function was described in another paper,^17^ the comparison of 246 CCS at mean time of 21.7 years after diagnosis and 211 matched controls, proved all key RV parameters such asRV *S*′ velocity, TAPSE, and RV free wall strain were significantly lower in the CCS group. This is in concordance with our results, however the time difference since cancer therapy to follow‐up examination was lower in our group. The findings described above may implicate that the impairment of cardiac function worsens with time, from subtle changes detectable by CMR of 3D‐RT echocardiography to a decrease of tissue Doppler velocities. The occurrence of arrhythmias in CCS was described in a large study by Armstrong et al.^18^ on more than 10,000 CCS and 3159 healthy siblings as controls, with median follow‐up of 25.6 years. The cumulative incidence of arrhythmias was 1.3% in CCS and 0.4% in healthy siblings (*p* < 0.001). The authors also proved negative cumulative effects of anthracyclines and radiotherapy, mainly in the occurrence of coronary artery disease. The worsening influence of the combination of radio‐ and chemotherapy was also significant in the incidence of valve diseases, heart failure, and arrhythmias. In our cohort, there were 46 (47%) subjects who received both chemo‐ and radiotherapy, but we did not find any significant difference in tissue Doppler parameters or arrhythmia occurrence between subgroups with or without the administration of anthracyclines.

Clinical perspectives: Based on the abovementioned results, we suggest the late cardiotoxic effects gradually worsen with time, affecting first the left ventricular diastolic function with simultaneous impairment of the right ventricular systolic parameters. The cardiotoxic effects may be potentiated by concomitant use of radiotherapy. Furthermore, long‐term follow‐up in adulthood is needed and regular echocardiographic controls are still the first‐choice method, with the addition of other methods such as cardiac MRI and ECG Holter monitoring in relevant cases.

Study limitations: Our study was only single‐center with a limited number of subjects and the follow‐up period was relatively shorter in comparison with other studies. However, we have been continuing to enroll subjects and further follow‐up is planned. In addition, the 24‐h ECG Holter monitoring is relatively short to detect all potential arrhythmias, however it is easier for patients to undergo.

In conclusion, current therapeutic protocols in children's cancer diseases, including chemo‐ and radiotherapy, cause initially subclinical impairment of cardiac function, measurable by tissue Doppler imaging.

## CONFLICT OF INTEREST

The authors declare no conflict of interest or industrial funding or relationship.

## AUTHOR CONTRIBUTIONS

All authors contributed to this manuscript: VK, RP, TK, and VB (V.Bajciova) prepared the conception and design of the study, and data analysis, LO and VB (V.Bednarova) contributed on drafts of the manuscript, JM did the statistical analysis and wrote statistical section of the manuscript. VK completed the final article and RP and TK revised it. All authors read and approved this manuscript and declare they have no conflict of interest.

## ETHICS STATEMENT

The study was approved by Ethics committee of St. Anne's University Hospital, Brno, Czech Republic, Reg. No. 15V/2018. All subjects gave their informed consent before enrollment into study according to Declaration of Helsinki.

## Data Availability

Data are available from authors upon reasonable request.
